# Vascular Stents Coated with Electrospun Drug-Eluting Material: Functioning in Rabbit Iliac Artery

**DOI:** 10.3390/polym12081741

**Published:** 2020-08-04

**Authors:** Konstantin A. Kuznetsov, Ivan S. Murashov, Vera S. Chernonosova, Boris P. Chelobanov, Alena O. Stepanova, David S. Sergeevichev, Andrey A. Karpenko, Pavel P. Laktionov

**Affiliations:** 1 Institute of Chemical Biology and Fundamental Medicine, Siberian Branch, Russian Academy of Sciences, 630090 Novosibirsk, Russia; vera_mal@niboch.nsc.ru (V.S.C.); boris.p.chelobanov@gmail.com (B.P.C.); lebedeva@niboch.nsc.ru (A.O.S.); lakt@niboch.nsc.ru (P.P.L.); 2 City Clinical Hospital no. 1, 630047 Novosibirsk, Russia; 3 Meshalkin National Medical Research Center, Ministry of Health of the Russian Federation, 630055 Novosibirsk, Russia; ivmurashov@gmail.com (I.S.M.); d_sergeevichev@meshalkin.ru (D.S.S.); andreikarpenko@rambler.ru (A.A.K.)

**Keywords:** paclitaxel, drug-eluting stents, 3D matrix, polycaprolactone, HSA, electrospinning

## Abstract

A stenting procedure aimed at blood flow restoration in stenosed arteries significantly improves the efficiency of vascular surgery. However, the current challenge is to prevent neointimal growth, which reduces the vessel lumen, in the stented segments in the long run. We tested in vivo drug-eluting coating applied by electrospinning to metal vascular stents to inhibit the overgrowth of neointimal cells via both the drug release and mechanical support of the vascular wall. The blend of polycaprolactone with human serum albumin and paclitaxel was used for stent coating by electrospinning. The drug-eluting stents (DESs) were placed using a balloon catheter to the rabbit common iliac artery for 1, 3, and 6 months. The blood flow rate was ultrasonically determined in vivo. After explantation, the stented arterial segment was visually and histologically examined. Any undesirable biological responses (rejection or hemodynamically significant stenosis) were unobservable in the experimental groups. DESs were less traumatic and induced weaker neointimal growth; over six months, the blood flow increased by 37% versus bare-metal stents, where it increased by at least double the rate. Thus, electrospun-coated DESs demonstrate considerable advantages over the bare-metal variants.

## 1. Introduction

Cardiovascular diseases are the leading cause of mortality worldwide, being responsible for 17.3 million lethal cases annually with forecasted increase to over 23.6 million deaths by 2030 [1]. Obliterating atherosclerosis accounts for up to 90% of all cardiovascular diseases. Rapid development of balloon angioplasty and stent implantation for restoration of the occluded vascular lumen, constant improvement of the tools for delivery and the materials used for stents, and improvements of their design have made it possible to fundamentally refine the arterial patency and reduce the rate of open surgery. Nonetheless, restenosis of the stented segment of an arterial wall in the long run remains a serious problem in endovascular surgery [2]. In terms of angiography, restenosis is defined as the stenosis of ≥50% of the vessel diameter in the late postimplantation period. From a clinical standpoint, restenosis is associated with relapse, namely, progression of the ischemia in the region of the stented vessel’s blood supply, which requires repeated surgery.

The rate of restenosis after balloon angioplasty during 5.9 ± 1.3 months is approximately 32% versus 18% for bear-metal stenting [3]. Thus, the use of bare-metal stents (BMSs) allows the rate of restenosis to be almost halved compared to balloon angioplasty; yet, it fails to completely resolve the problem.

The stent deployment into the vessel lumen itself induces mechanical stretching, frequently with rupture of the internal elastic membrane, crushing of the plaque, blood cell response to tissue proteins, and tissue response to foreign material, which in turn initiates inflammation followed by regeneration of the lesion. Endothelial and smooth muscle cells are involved in the regeneration as well as blood platelets and monocytes, which secrete cell growth and chemotaxis factors and cytokines [4,5]. Restenosis is the result of neointimal hyperplasia, which develops via the proliferation and migration of smooth muscle cells during the first month after stenting and the degree of which is determined by the degree of vessel wall lesion [6,7]. Note that restenosis develops on the background of atherosclerosis, and the pathogenesis of concomitant diseases, in particular, diabetes mellitus, also contributes to its development [8]. To prevent and inhibit restenosis, it was proposed to coat stents with drugs possessing cytostatic and/or anti-inflammatory properties. On the one hand, drug-eluting stents (DESs) remodel the affected vessel region, and on the other hand, they provide local drug delivery. Drugs with a cytostatic/cytotoxic effect belonging to the limus family compounds and paclitaxel are most frequently used for inhibition of neointimal proliferation [2]. These drugs are immobilized on the stent surface at different concentrations, both individually or in mixtures with different carriers [9].

One of the most widespread approaches is the use of relevant drugs in combination with biodegrading or biostable polymers used for coating stents [10,11]. On the one hand, such polymeric coatings are asserted to improve the delivery of a cytostatic to the vessel wall, but on the other hand, they can lead to adverse events. Biodegradable coatings involving polyhydroxyalcanoates may lose adhesion during both stent deploying and biodegrading. Fragmentation and biodegradation of the coating leads to distal embolization and increases the risk of local thrombosis and microcirculatory failure. Cases of cracking and inhomogeneity of the polymeric layer in Taxus (paclitaxel) and Cypher (everolimus) stents even before implanting have been reported, which leads to the abovementioned complications [12]. The hydrolysis products of polyhydroxyalcanoates acidify the ambient medium, which can additionally activate inflammation [13].

Independently of the coating material, it is applied onto the stent strut (with a thickness of ~200 µm and total area not exceeding 20% of the vessel wall area after stent placement) and is delivered to the contact region between struts and tissue. In order to attain cytotoxic concentrations of drugs in the tissue located between the stent struts, high doses of the drug are necessary, which in turn can critically slow down the cell activity, cause aneurysms, and/or stent “slopping” [14,15]. In addition, a considerable part of the drug enters the systemic flow [16].

The surface between the struts remains free from the cytostatic, while the fragments of destructured tissue (plaques) not held by the stent struts may enter the blood flow or, on the contrary, may dishevel and initiate clotting and neointimal growth. To prevent these events, the Inspire MD Inc. stents were designed; these stents are covered with a MicroNet—biostable mesh woven from a single strand are declared by manufacturers to effectively prevent neointimal growth interfering with the penetration of external tissues [17].

Another separate and yet unresolved problem is the control of drug release. The concentration of the preparation released from the coating to the vessel cell should prevent neointimal growth both at the early stages of stent placement, when an active inflammatory response develops, and in the long run, when it is necessary to inhibit the excess cell growth in the immediate vicinity of the stent. Note that it is undesirable in the long run to inhibit the growth of more distant cells of adjacent tissues to prevent the initiation of aneurysm development.

It is noteworthy that DESs, according to data from the latest multicenter randomized studies, do not display significant advantages over bare-metal stents [18], while the rate of restenosis during the observation period (12–15 months) is 6.7–12% [19,20,21].

To solve the above-listed problems, it was proposed to cover stents with 3D matrices formed by electrospinning, which has been shown to be successfully used for the production of various cardiovascular implants [22,23,24]. The experimental esophageal and vascular stents thus coated were manufactured [25,26,27]. In particular, the PK Papyrus (Biotronik, Berlin, Germany) stent is used in clinical practice; in this stent, the electrospun polymeric coating acts as a graft for hermetic sealing of coronary artery lesions [28,29]. The use of electrospinning for coating stents indeed looks very attractive as this method makes it possible to produce coatings from fibers with a diameter of several tens of nanometers to several micrometers, fabricate such fibers of synthetic and natural polymers or their mixtures, introduce various drugs into the fibers, and form layers with the help of successive or concurrent production of different types of fibers [30,31,32,33,34]. The coating thus produced stretches during stent strut expansion and provides a mechanical support for the vessel tissue (as in the InspireMD stents MicroNet [17]). It prevents blood loss in the case of vessel wall rupture and, additionally, can have the required kinetics of drug release [35]. By forming multilayered coating with drug-“retaining” layers, it is possible to provide a vector delivery of the drug to the vessel wall, thereby decreasing its release into the systemic flow. Thus, the use of stents with electrospun coatings is a promising approach to improve the properties of vascular stents.

Previously, we studied 3D matrices produced by electrospinning from different blends of polycaprolactone with cytostatic drug paclitaxel by physical and chemical methods in order to find the best material for coating of vascular stents [35]. Paclitaxel release in phosphate-buffered saline (PBS) and human serum was studied using tritium-labeled drug. In these experiments, we found that 3D matrices produced from a solution of polycaprolactone with human serum albumin and paclitaxel in hexafluoroisopropanol with 3% dimethyl sulfoxide were characterized by two-phase drug release with potential to support toxic concentration of paclitaxel against vascular wall myocytes for at least three months [35].

Here, we describe the procedure of coating a stent’s outer surface with a 3D matrix produced by electrospinning, placement of this stent onto a balloon catheter, and comparative data on the functioning of the stents with and without the coating when implanted to the rabbit iliac artery.

## 2. Materials and Methods

### 2.1. Reagents

The following reagents used in the current study were from Sigma-Aldrich, United States: polycaprolactone (PCL, Mn 70.000–90.000), human serum albumin (HSA), 1,1,1,3,3,3-hexafluoroisopropanol (HFIP), paclitaxel (PTX), dimethyl sulfoxide (DMSO); formalin, PBS, (3-aminopropyl)triethoxysilane, glutaraldehyde, poly-l-lysine, 2-methoxyethyl acetate, benzoyl peroxide, dicyclohexyl phthalate, and hexamethyldisilazane (HMDS).

Atropine (Dalkhimpharm, Khabarovsk, Russia), XylaVet (Pharmamagist, Budapest, Hungary), Zoletil-100 (Virbac Sante Animale, Kansas, France), 0.9% NaCl solution (Krasafarma, Krasnoyarsk, Russia), 10% Betadine (EGIS Pharmaceuticals PLC, Budapest, Hungary), Heparin (Sintez JSC, Moscow, Russia), 50 mg/mL sodium thiopental (Sintez JSC, Moscow, Russia), and aluminum spray (NicoVet, Berlin, Germany; no. 018-075) were used for in vivo experiments.

LR White Resin (cat. no. 14383-UC, London Resin Company, Ltd., United Kingdom), hematoxylin, VitroGel medium (Biovitrum, St. Petersburg, Russia), and eosin (Bio-Optica, Milan, Italy) were used for histological examination.

### 2.2. Electrospinning of Drug-Eluting Coating and 3D Scaffolds

The electrospinning solutions were prepared using stock solutions of 9% PCL and 1% HSA in HFIP. The HSA concentration in matrices is given as weight percentage (wt/wt) of the total matrix weight. Paclitaxel was dissolved in HFIP or DMSO and added to the matrix (~0.46 μg/cm^2^). DMSO (3% *v*/*v*) was added to the solution of polymers. The 3D matrices of ^3^H-PTX were produced using a custom-made electrospinning device with an airproof chamber and exhaust HEPA filter equipped with a Spellman SL 150 (30 kV; Spellman, United States) power supply. A polished stainless steel rod with diameter of 1.0 mm and length of 30 mm with previously installed metal stent (min/max diameter 2.0/3.5 mm; Angioline, Novosibirsk cit, Russia) was used as an electrode (stent was settled using conical expansion and settled down with a cylindrical crimping device followed by rolling over a silicone rubber sheet). Coating of 150–180 µm was applied onto the stent at a feed rate of 1.2–1.4 mL/h, capillary–collector distance of 19–20 cm, voltage of 23–25 kV, collector rotation speed of 300 rpm, temperature of 23–25 °C, and humidity of 25–35%. One stent required 0.23 mL of the polymer solution. After fabrication, the coated stent was removed from the collector, vacuum-dried at 10 Pa for 12 h, and stored in sealed zip-lock polyethylene containers at 4 °C.

Before installation to a balloon catheter, the coated stents were put on a rod with a diameter of 1 mm with a conic junction of 15° from 1 to 1.5 mm ending with a blunt end. The stent was pushed from this end onto the balloon catheter preliminary folded as an “asterisk”. After the installation, the stent was additionally rolled in for sealing on the delivery device simultaneously with evacuation of the balloon catheter with a syringe manometer. The delivery device with the installed stent was sterilized with ethylene oxide in standard conditions supplied by the manufacturer using 3M Steri-Vac 5XL (3M, Old Saybrook, Connecticut state, USA).

Scaffolds for blood tests were produced in the same conditions using a drum collector [36] and different compositions of electrospinning solution as described [35]. Disks of 10 mm were excised from matrices by die cutting and used as an individual probe.

### 2.3. Characterization of Electrospun Coating

#### 2.3.1. Hemolysis Assay

The test was executed as recommended by ISO 10993-4 and as previously described [36]. Briefly, anticoagulated blood was diluted twice with PBS and incubated with 10 mm discs for 1 h. After incubation, cells were pelleted by centrifugation (1000 rpm, 10 min), and optical absorbance of the supernatant was measured at 545 nm. The percentage of hemolysis was calculated as [(ODsample − ODneg)/(ODpos − ODneg)] × 100. Tests were performed in three replicates for all types of scaffolds.

#### 2.3.2. Adhesion of Platelets or Blood Cells on the Materials

The test was done as previously described [36] using scaffolds installed in a 48-well plate with platelet-rich plasma or citrate blood. After incubation, the matrices were washed twice with PBS to remove nonadherent platelets or blood cells and fixed in 2% formaldehyde in PBS for 30 min. Fixed blood cells were dehydrated using a graded ethanol series (50%, 70%, 90%, and 100%) and then transferred to mixtures of ethanol and HMDS in a ratio of 1:1, then 100% HMDS. After drying and sputter coating with 10 nm gold/palladium layer, matrices were analyzed by scanning electron microscope EVO 10 (Carl Zeiss AG, Germany). Tests were performed in three replicates for all types of scaffolds.

#### 2.3.3. Study of the Elongation of the Coating

Coated stents installed onto the balloon catheter as previously described before and after balloon expansion were studied. Stents were removed from the balloon, fixed on a sample stand using double-sided carbon tape, and coated with 10 nm gold/palladium layer. The morphology of the outer and inner surfaces of the electrospun material was studied using scanning electron microscope EVO 10 (Carl Zeiss AG, Germany).

### 2.4. Experimental Animals

In total, 36 laboratory Chinchilla rabbits with an average age of 6.8 ± 0.15 months and an average bodyweight of 4.50 ± 0.5 kg were used in this work. All manipulations with animals complied with the European Convention of laboratory animal protection [37] and were approved by the ethics committees of NMRC MH RF (NCT02255188 from 16.01.2014). The animals were kept under stationary vivarium conditions: natural lighting, standard diet, water ad libitum, and one rabbit per cage. According to the relevant sanitary, epidemiological, and veterinarian regulations, the animals were quarantined for at least 2 weeks after entering the vivarium before the experiments.

The stents were implanted to the iliac artery of rabbits under sterile conditions. In total, six coated DESs and six BMSs were implanted at each observation period (1, 3, and 6 months). After the surgery and after awakening from anesthesia, each animal was placed into a cage with the necessary postsurgery care. During the first 7 days, nonsteroidal anti-inflammatory preparations were injected intramuscularly to prevent the pain syndrome, and an antiplatelet drug (aspirin daily at a dose of 75 mg) was administered orally over the observation period to prevent thrombosis.

On completion of the observation period, the studied material was explanted under anesthesia (laparotomy, isolation, and excision of the iliac artery with the stent).

Immediately after sampling the experimental specimens, the animals were euthanized before awakening with sodium thiopental (50 mg/kg bodyweight).

### 2.5. Procedure of Experimental Stent Implantation

Atropine (0.1% at a dose of 0.1 mg/kg) was intramuscularly administered as a premedication (to reduce the secretion of salivary and bronchial glands). The mixture of XylaVet (15 mg/kg bodyweight), Zoletil (15 mg/kg bodyweight), and 0.9% NaCl solution was used for anesthesia. The anesthetized animal was fixed on the surgical table in a supine position. The hair was shaved from the right neck side.

The surgical area was sterilized with an antiseptic (10% Betadine); the carotid artery was accessed via the carotid triangle. Heparin (100 U/kg) was injected into the internal jugular vein.

The carotid artery was punctured according to Seldinger to install introducer 5F (with guiding catheter and sheath needle 5F, 0.035″ 18G; cat no. AB5132; Angioline, Novosibirsk, Russia). A hydrophilic guidewire (Abbott Vascular Guidewires, Hi-Torque Pilot 200 Guide Wire, cat. no. 1010482-HJ; 0.014″ × 190 cm) with a 5F guiding catheter (Merit Medical, 5F PERFORMA MW2, 65 cm, 038′, 1.17 mm internal diameter) was used to deliver the stent to the terminal abdominal aorta. Angiography allowed the anatomy of the iliac artery to be assessed. The guiding catheter was extracted. The examined stent (a balloon catheter with a Sinus stent RxPTCA, cat. no. CP08300, 0.014″ × 190 cm; inflation device with a Klik Y connector, guide introduction tool, and guide rotation tool no. AV6301; volume, 20 mL; pressure, up to 30 atm; Angioline, Novosibirsk, Russia) was positioned and implanted using the prepared delivery system. The accuracy of stent positioning was controlled by angiography, and the introducer and guide were removed. The puncture site was sutured (7/0), the wound was sutured in a layerwise manner, and sutures were treated with aluminum spray.

In total, 18 control BMSs and 18 DESs coated by electrospinning were implanted.

### 2.6. Intravital Estimation of Stent Patency

The patency of the stented artery was assessed using dynamic ultrasound examination 1, 3, and 6 months after the implantation. No complications were recorded during the observation period.

The mean linear blood flow velocity (LBFV) in the stent was estimated using a Vividi sonography system (GE Medical Systems, Tirat Hacarmel, Israel). The velocity was measured immediately upstream of the proximal end of the stent, in its proximal part, in its central part, in its distal part, and immediately after its distal end to assess the presence and rate of the flow.

### 2.7. Histological Examination of the Vessel Wall in the Arterial Segment with Implanted Stents

Visual examination for stent deformations and changes in the adjacent tissue was performed intraoperatively during sampling. After the autopsy, the aorta abdominal region and iliac arteries with the tested stent were excised, and the stented segment of the artery was cut off at a distance of 1 cm from the stent boundaries.

The explanted stents were washed with physiological saline (5 mL) using an insulin syringe to remove the blood cells and fixed in 10% formalin in phosphate buffer.

A SteREO Discovery V12 (Carl Zeiss, Germany) stereomicroscope was used for survey to assess the outer and inner diameters/areas of the lumen of the examined stents.

For histological examination, the explanted stents with a fragment of the artery were dehydrated and embedded in LR White Resin (cat. no. 14383-UC, London Resin Company, Ltd., United Kingdom) according to the manufacturer’s protocol with a minor modification: the resin was supplemented with benzoyl peroxide blend with dicyclohexyl phthalate (2 g per 50 mL resin) for initiation of polymerization and polymerized at 55 °C for 48 h. The sections (thickness, 8 µm) were made using a Microm HM-550 (Thermo Fisher Scientific Inc., United States) microtome equipped with a tungsten carbide knife (cat. no. UWCDIS, ProSciTech, Ltd., Australia), spread on glass slides (Biovitrum, Russia) modified with (3-aminopropyl)triethoxysilane as described in [38]. For fixation, the sections were incubated under pressure in 1% glutaraldehyde and 0.33 mg/mL poly-l-lysine for at least 24 h in a humid atmosphere and then for at least 24 h in the open air at 42 °C. The sections were deplasticized in 2-methoxyethyl acetate as earlier described [39].

The sections were stained with hematoxylin and eosin to determine the main structural components of the stent implantation region, namely, cell filling, noncellular structures, neointimal proliferation, and signs of inflammation.

The stained slides were dried and immersed into a Vitrogel medium under a cover glass. AxioLab A1 with the Zen 2 Blue Edition software package (Carl Zeiss, Germany) was used for microscopy.

### 2.8. Data Statistical Processing

Microsoft Excel 2010 was used for the initial data accumulation and processing, and Statistica 7 for Windows 7 (StatSoft Inc., Tulsa, Oklahoma state, United States) was used for final processing. The differences were regarded as statistically significant at *p* < 0.05.

## 3. Results

**Figure 1:**
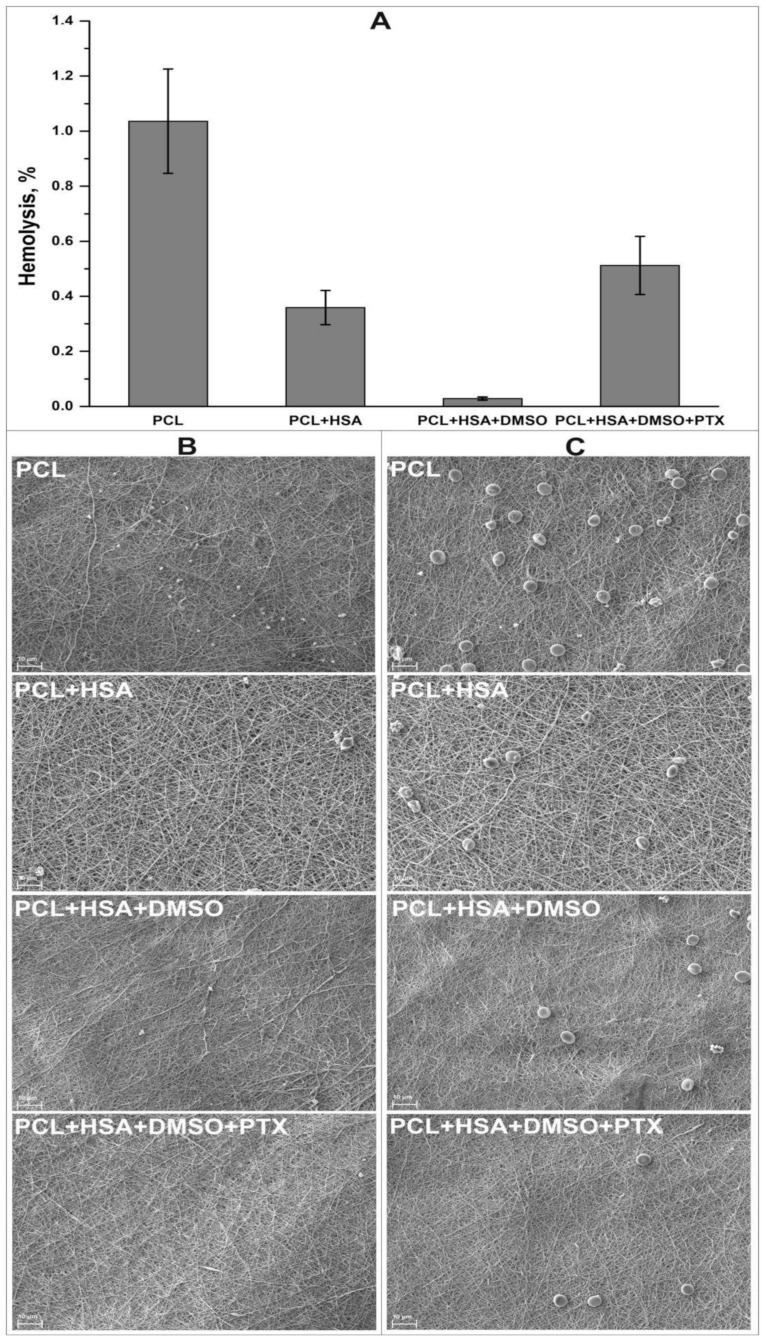
Hemocompatibility of stent coating and a control scaffolds. Panel **A** represents a hemolysis rate induced by different scaffolds. SEM images of surface of the scaffolds after incubation with platelet-rich plasma (panel **B**) or citrate blood (panel **C**); magnification, ×2000.

In this study, we coated the bare-metal stents with a 3D matrix produced by electrospinning. The physicochemical properties of such 3D matrices and their ability to release PTX were studied earlier [35]. The material consists of fibers with a diameter of 0.567 ± 0.091 µm with pores of 5.72 ± 2.42 µm and is freely permeable for liquids. As has been shown, the coating deformation when placing the stent falls into the region of plastic deformation, i.e., the coating does not add the strain on the stent, while the residual load does not exceed 5% of the load on the vessel wall [35]. Electrospun 3D matrices produced from PCL and protein-enriched PCL were previously shown as hemo- and biocompatible materials [40,41]. Incubation of the matrices with platelet-rich plasma or citrate blood demonstrated that cell adhesion was dependent on the chemical composition of the fibers. Minimal number of adhered cells was observed for PCL+HSA+DMSO+PTX coating compared with the control scaffolds (Figure 1, panels B and C). The results of hemolysis test showed moderate hemolysis of the stent coating compared to the tested scaffolds (Figure 1, panel A).

Moreover, nonbiodegradable covering of BMS has been shown to be beneficial compared to biodegradable thanks to prevention of direct contact between metallic struts and vascular wall [42], and PCL has been offered for stenting application [43]. In addition, the matrices fabricated of PCL with HSA and PTX in HFIP with 3% DMSO had a biphasic kinetics of PTX release; moreover, the cytotoxic PTX was retainable in the vessel cell for at least 27 days, which, according to the literature data, is enough to prevent the neointimal growth in the near term and long run [44]. The stents were covered with these particular coatings.

**Figure 2:**
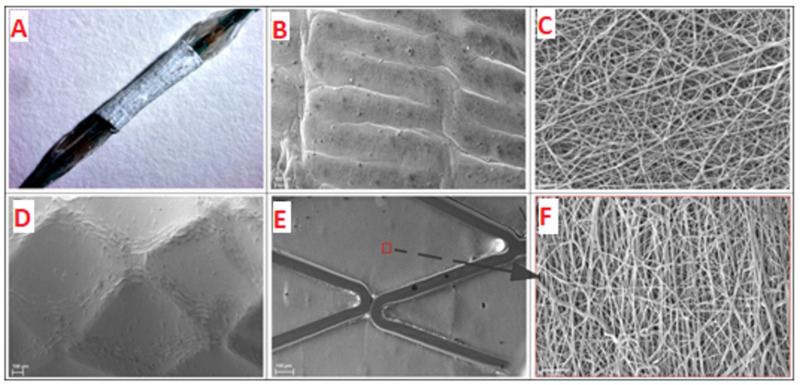
A stent with the electrospun coating installed onto balloon catheter (**A**). Image obtained with SteREO Discovery V12 microscope (Carl Zeiss, Germany). SEM images of coating deformation after installation onto balloon catheter before (**B**,**C**) and after balloon expansion (**D**–**F**). Panels **B**,**C**,**D** show outer surface of electrospun coating; panels **E** and **F** show inner surface of electrospun coating (**B**,**D**,**E** panels—×149 magnification; **C**,**F** panels—×5000 magnification).

**Figure 3:**
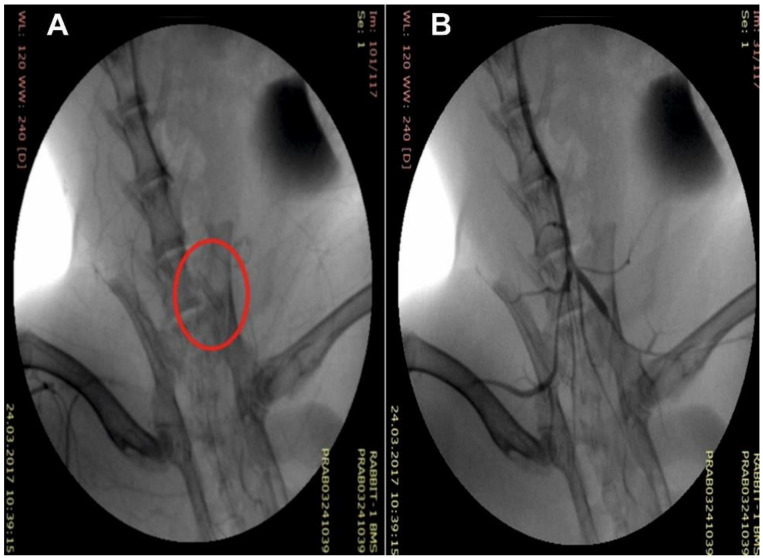
Intraoperational view of the stent with tissue engineered coating (C arm): (**A**) an implanted stent and (**B**) its angiographic control.

Because the coating produced by electrospinning shrinks and the region of its elastic deformation amounts to approximately 8–10% [35], the procedures described in Materials and Methods allowed for removal of the coated stent from the electrode and its shrink-fitting on the balloon (Figure 2A), providing its stability during delivery to the iliac artery and precise positioning at the target site (Figure 3). When pressing on the coating of the balloon catheter, slight deformation was observed, which did not affect the orientation of the electrospun fibers (Figure 2B,C). Stent expansion led to deformation of the covering accompanied by alignment of the fibers and small pressing of the coating inward between the stent struts (Figure 2D–F). It should be noted that folds of the material on the surface of the struts were obviously related to a small “reverse” deformation of the stent after its expansion (Figure 2D).

When assessing the state of blood flow region after the implantation of the stents, neither limb gangrene nor any signs of trophic disturbance in the limbs was observable. None of the experimental rabbits displayed intermittent lameness, and the skin of their hind limbs and tail was of normal color.

The patency of BMSs and DESs was 100% over the observation period.

The LBFV was measured at five control points (see Materials and Methods); as this velocity differed by no more than 3–5%, the values were averaged. The values in stents of different groups are listed in Table 1. The LBFV in the group with BMSs by the end of the observation period (6 months) was 0.69 ± 0.01 m/s versus 0.42 ± 0.01 m/s in the group of DESs; the mean LBFV (without any surgical intervention) was 0.3 ± 0.01 m/s.

Thus, the blood flow velocity in the vessels with BMSs increased by 25% one month after implantation; an additional 20% three months after implantation, and an additional 27% six months after implantation. This means the LBFV more than doubled over the observation period with an evident trend of a linear growth. As for the DESs, the blood flow velocity did not increase one month after implantation, increased approximately by 23% during the next two months, and grew by only 7% during the next three months. As the blood flow velocity reflects the general effective vessel lumen, the LBFV data suggest a rapid decrease in the effective section of the lumen after BMS placement and a considerably slower (with the trend of stabilization) decrease in the effective section in the case of DESs.

**Figure 4:**
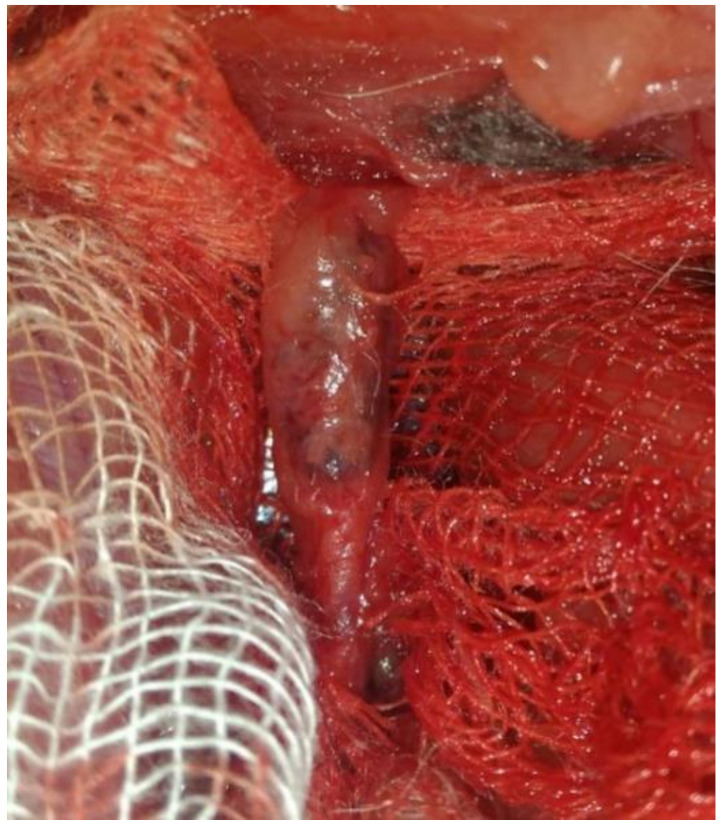
Explantation of the iliac artery with implanted stent. Image obtained using OPMI Pico microscope (Carl Zeiss, Oberkochen, Germany).

Figure 4 shows a typical image of the stented vessel after autopsy and isolation of the vessel. Diastasis, purulence, or any other complications of the postoperative wound were unobservable. Arteries of “access” through which the delivery system was introduced did not show signs of thrombosis and obliteration.

**Figure 5:**
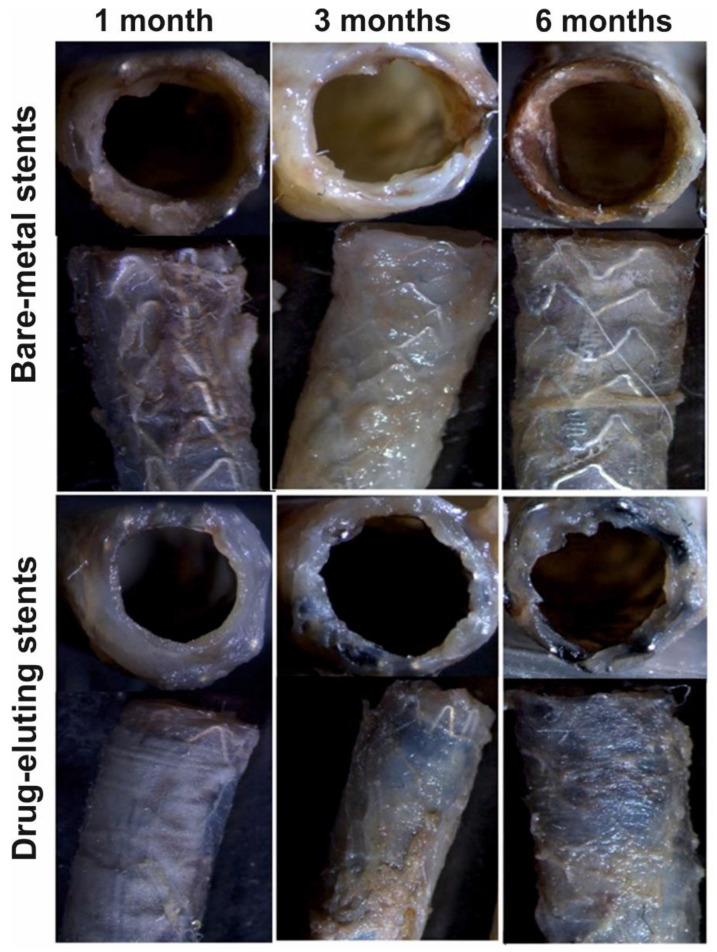
Survey microscopy of the vessels with implanted stents at different time points of observation. Images obtained using SteREO Discovery V12 microscope (Carl Zeiss, Germany).

**Figure 6:**
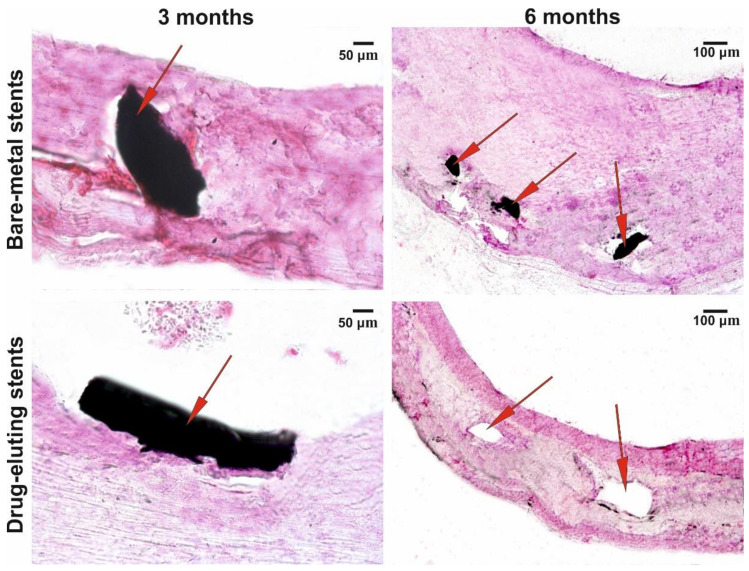
Microscopy of the cross sections of implants at different observation points. Staining with hematoxylin and eosin; AxioLab A1 with the Zen 2 Blue Edition software package (Carl Zeiss, Oberkochen, Germany); magnifications, ×100 and ×400; arrows denote stent’s struts or the sites where the struts are located.

A native vessel adventitia without any changes was observed at the stage of sampling. Visually, no changes in the contact between the adventitia and adjacent tissues were observable in the stented segment of the iliac artery, which suggests the absence of rejection response, inflammation, and peritoneal process. Any signs of infection or pronounced inflammation and hematomas were absent in both the stented region and neighboring tissues (Figure 5). Macroscopic examination did not find any visible deformations of the wall and coloration in the vessels with implanted experimental stents (DESs). Sharp boundaries between the stent regions and native vessel were also absent (Figure 6). The stent coating was located in the same position on the stent as in the preoperative stage, and no ruptures of corrugation of the coating were observed.

The vessels with BMSs displayed an evident trend of decrease in the thickness of the vessel wall in the region of contact with the stent’s struts, which was more pronounced after six months of stent functioning. However, this situation was unobservable in the vessels with DESs; presumably, this is associated with a more uniform distribution of the load on the vessel wall (as the coating distributes the load of the strut–wall contact region to the space between the struts).

A growth in the fibrous tissue with different degrees of maturation (neointima) was observed in the vessels with BMSs as early as three months after implantation, which was even more pronounced after six months (Figure 6). As for the vessels with DESs, weak penetration of the stent’s struts into the intima was observed three months after implantation and the formation of a dense tissue layer contacting the blood flow was observed six months after the surgery. Fibroblasts and the surface layer of endothelial-like cells were identifiably at a higher resolution. The neointima in the arteries with BNSs were significantly thicker compared with the vessels with DESs and had a structure more similar to the sclerosed regions of the vessel wall with almost indistinguishable collagen fibers.

## 4. Discussion

The consolidated data [18] suggest that, with respect to restenosis, there is no significant difference between BMSs and DESs in the long run of over six years [2]. However, when considering the observation period of up to five years, adverse clinical manifestations, rate of restenosis, and mortality for DESs are lower compared to BMSs [9]. The drug concentration in the stent coating and the way of their immobilization there, which influence the rate drug release, are fundamental questions. Most frequently, stents are ultrasonically coated with PTX at a dose of 3 µg/mm^2^ [45]. In particular, the Zilver PTX stent, which is widely used in clinical practice for the peripheral arteries of the lower limbs, is produced according to this technique [46]. The use of such high concentrations of a cytostatic causes several adverse events, such as tissue necrosis, decrease in the vascular wall strength with subsequent aneurysm development [19], destruction of coating and distal embolism caused by its fragments [47], local thrombosis, and development of local noninfectious inflammatory focus [48], which has induced the recommendations against their use [18].

The PTX concentration in 3D matrices used in this study for coating metal stents was 0.46 µg/cm^2^ (which is 650 times less than in [45]). The PTX concentration toxic for smooth muscle cells is ~10 nM [48]. Taking into account the initial boost release of PTX followed by 1% daily release, its diffusion through the arterial wall, and interaction with the arterial wall proteins, PTX is toxic for the vascular wall myocytes for at least 1–3 months [35].

Our data demonstrate the efficiency of DESs coated with PTX by electrospinning. As shown in the ex vitro experiments, the coating was not apt to adhesion of platelets and other blood cells and induced hemolysis to a much lesser extent than allowed by ISO 10993-4. The LBFV in DESs differed considerably from that in BMSs. During the first month, we did not observe an increase in LBFV at all, most likely because of efficient inhibition of neointima-forming cells by the released PTX. In the next two months, the effective section of the stented segment somewhat decreased; however, the decrease was minimal (7%) during the next three months, suggesting stabilization of the neointimal formation.

The histological examination suggests that a certain change in the DES effective lumen three months after implantation is associated with a decrease in the concentration of the cytostatic, which ceases to inhibit the growth of neointima-forming cells. Our data match well with the data on PTX release [35]: PTX cytotoxic concentration was s maintained in the vessel wall for one month (no changes in the wall); suboptimal PTX concentration was retained for additional 2–3 months (small neointimal growth); and the newly formed tissue grew after completion of the cytostatic release. In the next three months, the layer of neointima was formed in the stented segment and a connective tissue capsule around it (Figure 6); presumably, their formation had a positive effect on stabilization of the lumen of the stented arterial segment. Note that the neointima in DESs considerably differed in its structure from that in the vessels with BMSs; most likely, this is explainable by the fact that it had been formed already after the acute stage of the inflammation caused by stent implantation.

The coating electrospun onto stents provides a mechanical protection of the bloodstream from penetration of the vascular wall cells and alignment of the inner surface (Figure 2D), similar to the Inspire MD stents covered with a MicroNet [17]. Moreover, the electrospun matrix displays a high mechanical performance, which enhances the distribution of the load onto the arterial wall. Figure 5 and Figure 6 demonstrate the preservation of the vessel wall structure along its length, both when contacting the struts and between them. In addition, the coating makes it possible to avoid direct contact between the vessel wall cells and metal, thereby increasing stent biocompatibility and decreasing inflammation at the stent–tissue interface, which is inevitably present because the metal surface is foreign and has different mechanical properties. The layer of 3D matrix acts as a buffer and can level the undesirable contacts and mechanical misfit of the stent material and living tissue.

Note that manifold variants of DESs coated by electrospinning are described in the relevant literature, including stents for tracheal regeneration [26], electrospun covered duodenal stents [27], and esophageal stents intended for restoration of the esophageal patency in benign esophageal structures [25]. In the last case, polycaprolactone coating with PTX was used; the corresponding animal experiments have shown that DESs with this coating reduces the smooth muscle cell proliferation and postpones restructure formation.

DESs coated by electrospinning with biostable or biodegradable polymers able to release anti-inflammatory preparations, cytostatics, anticoagulants, ligands for endothelization, and so on have been used to restore the vascular lumen [17,23,28,47,49,50]. The authors of all papers note that the stents of this type are most promising healthcare products, which improve endothelialization and reduce inflammation and clotting depending on the fillers used.

## 5. Conclusions

In summary, electrospun-coated DESs demonstrate considerable advantages over BMSs as indicated by sonography, survey microscopy, and histological examinations. Impact of mechanical protection of the wall and vessel lumen versus cytostatic action of electrospun coating to the efficacy of DESs has to be determined in further studies. Most likely, ex vivo investigation of the kinetics of tritium-labeled PTX can give a deeper insight into its mechanism of action and effect on neointimal growth. Presumably, slowed PTX release kinetics or its increased concentration may have a positive effect and allow for more efficient inhibition of neointimal growth. In order to evaluate the role of mechanical support of the stent coating, a thorough comparative study of stents coated with drug-eluted and drug-free materials is necessary. Evidently, a more comprehensive study into the function of the stented arterial segment in a prolonged follow-up is necessary at the next stage of the study in order to assess the efficiency of DESs.

## Figures and Tables

**Table 1:** Linear blood flow velocity in the region of implanted stent at different time points of observation.

Stent Type	Observation Period, Month	V = ΣV_n_/n, m/s, *
	1	0.4 ± 0.011
*Bare-metal stent*	3	0.5 ± 0.012
	6	0.69 ± 0.011
	1	0.3 ± 0.005
*Drug-eluting stent*	3	0.39 ± 0.011
	6	0.42 ± 0.005

* The data are shown as the mean ± error of the mean.
